# General Labor Well-Being in Latin American Dentists during the COVID-19 Pandemic

**DOI:** 10.3390/ijerph19106317

**Published:** 2022-05-23

**Authors:** Teresa Evaristo-Chiyong, Manuel Antonio Mattos-Vela, Andrés A. Agudelo-Suárez, Ana del Carmen Armas-Vega, Juan Carlos Cuevas-González, Clarisse Virginia Díaz-Reissner, Ana Cristina López Torres, Cecilia María Martínez-Delgado, Manuel Amed Paz-Betanco, María Antonieta Pérez-Flores, Sylvia Piovesan-Suárez, Adriana Pistochini, Yajaira Romero-Uzcátegui

**Affiliations:** 1 Grupo de Investigación SAETA, Universidad Nacional Mayor de San Marcos, Lima 15081, Peru; tevaristoc@unmsm.edu.pe; 2 Facultad de Odontología, Universidad de Antioquia, Medellin 050010, Colombia; oleduga@gmail.com; 3 Universidad Central del Ecuador, Quito 170902, Ecuador; acarmas@uce.edu.ec; 4 Departamento de Estomatología, Universidad Autónoma de Ciudad Juárez, Ciudad Juárez 32320, Mexico; juan.cuevas@uacj.mx; 5 Facultad de Odontología, Universidad Nacional de Asunción, Asunción 001217, Paraguay; diazclarisse@gmail.com; 6 Facultad de Odontología, Universidad de Costa Rica, San José 11501, Costa Rica; cristlopez@gmail.com; 7 Facultad de Odontología, Universidad CES, Medellin 050021, Colombia; cmariamar@hotmail.com; 8 Facultad de Odontología, Universidad Nacional Autónoma de Nicaragua, León 21000, Nicaragua; manuel.paz@fo.unanleon.edu.ni; 9 Facultad de Odontología, Universidad de Concepción, Concepción 4030000, Chile; mperezf@gmail.com; 10 Facultad de Odontología, Universidad de la República, Montevideo 11600, Uruguay; sylvia.piovesan@gmail.com; 11 Facultad de Ciencias de la Salud, Universidad Maimónides, Buenos Aires C1405BCK, Argentina; pistochini.adriana@maimonides.edu; 12 Facultad de Odontología, Universidad de los Andes, Mérida 5101, Venezuela; yromero581@gmail.com

**Keywords:** working conditions, COVID-19, dentists, health surveys, stress, psychological, post-traumatic stress disorder

## Abstract

This study aimed to determine the general labor well-being of Latin American dentists according to sociodemographic characteristics during the COVID-19 pandemic. A cross-sectional study was conducted in a final sample of 2214 participants from 11 countries. A validated online questionnaire on general work well-being was used (data collection period from 1 June to 10 July 2021), containing two dimensions: psychosocial well-being and collateral effects. The sociodemographic characteristics of the dentists and their perception of the economic impact of the pandemic were also recorded. A multivariate linear regression analysis was performed (hierarchical regression model) to evaluate the joint effect of the explanatory variables on labor well-being and the changes in the variance between each model. A score of psychosocial well-being of 233.6 + 40.2 and collateral effects of 45 + 20.1 was found. Psychosocial well-being was associated with sex, country of origin, academic training achieved, type of dental activity, and perceived impact during the pandemic (*p* < 0.05). Somatization was frequently manifested through back pain (88.2%) and muscular tensions (87.2%). Women, those who worked 41 or more hours and had between 1 to 15 years of professional experience presented a greater collateral effect (*p* < 0.001). The impact of the COVID-19 pandemic a year and a half after it began on the labor well-being of Latin American dentists was evidenced with important interactions with social characteristics.

## 1. Introduction

The coronavirus (COVID-19) pandemic is spread by SARS-CoV-2 virus, mainly through saliva or respiratory fluids released when coughing, sneezing, talking, or singing [1,2]. It can be transmitted indirectly when the hands are in contact with contaminated surfaces and then take the virus to the face’s mucous membranes (eyes, nose, and mouth) [2].

Dentists, who are in direct contact with patients and exposed to oral fluids and blood, may be subjected to a possible high risk of contagion [3,4]. However, some more recent studies mention that COVID-19 frequency is lower as a result of implementing biosafety protocols. Cross-infection by exposure to oral cavity and airway microorganisms and viruses represents a high possibility of daily contagion [5]. Thus, the initial step in several Latin American countries was to restrict dentistry activities, limiting them to emergency and urgent treatments [6,7,8]. Activity has gradually resumed with a strict compliance of biosafety protocols, but the potential contagion risk caused dentists, especially those at higher risk due to their age or systemic conditions, to attend their practices less frequently or to stop attending all together. Dentists had to perform in uncertain, stressful, and worrisome circumstances, which disturbs their occupational health psychosocially (affecting emotions, skills, and expectations). If these effects persist, they will produce somatization, wear, and alienation [9].

Work well-being and life quality are influenced by work environment interests, values, and perceptions related to psychological, social, and behavioral factors [10].

Various psychosocial factors have been identified to have an impact on work well-being: attitude towards change, expectations, anxiety, anguish, lack of support from the direct superior, work overload, stressful environment, high emotional workload, insecurity in the workplace, etc. [11,12].

Research in Latin America indicates that confinement due to the COVID-19 pandemic harmed perceived stress and subjective well-being in dentists (general and specialists) and dental students [13,14,15], specifically finding an association with income level, having older adults in care, self-perceived level of concern regarding COVID-19, self-perceived health, and coffee consumption [13], as well as with sleep quality, several biosecurity measures, age, and perception of their health status [15], and with following preventive measures: concern about the pandemic, decreased economic income, and gender [14]. Italian dentists showed feelings of concern (70.2%), anxiety (46.4%), and fear (42.4%) in practicing their work activity during the COVID-19 pandemic. Additionally, 89.6% of them feared an uncertain professional future [8]. In Telangana (India), the average fear and anxiety score was high considering both symptoms (6.57 ± 2.07). No particular relationship was found between this result and demographic variables: age, sex, academic qualification, type of practice, years of professional experience, and place of residence [16]. In Israel, a high risk of psychological distress was found in 11.5% of a sample of dentists with a systemic condition background, caused by the fear of being infected by a patient and by having high emotional overload [17]. In British dentists, 77.2% were economically affected by the pandemic and 41.1% suffered a psychological impact [18].

Studies on stressful circumstances and emergencies show that in addition to the fear of infection, there are also feelings of frustration and boredom caused by the inability to cover basic needs (financial problems with psychological impact). In general terms, people that have lived through a quarantine during COVID-19 pandemic are more prone to suffer an acute psychological stress disorder due to all the prevention, isolation, and protection measures that must be taken [19]. Health professionals, and dentists in particular, must use personal protective clothing and equipment to avoid contagion [5]. However, this equipment generates discomfort when working and difficulty breathing, which leads to higher work stress and emotional overload [20]. Psychological stress is not only limited to the pandemic situation, but can be related to social, cultural, and environmental factors that vary in each country [21].

Faced with this scenario, dentists are exposed to many factors that affect their work well-being, especially in Latin American countries with health systems with limitations. Therefore, this problem needs to be studied to contribute to collaborative decision-making aiming to meet the work well-being needs of dentists and improve their quality of life, which, in turn, will result in better oral health care for the Latin American population [22].

This study aimed to assess the general work well-being of Latin American dentists according to their sociodemographic characteristics during the COVID-19 pandemic, since published studies in this regard are scarce.

## 2. Materials and Methods

### 2.1. Design, Population, and Sample

A cross-sectional study was conducted. The study population comprised dentists from eleven Latin American countries: Peru, Chile, Mexico, Colombia, Argentina, Ecuador, Uruguay, Venezuela, Paraguay, Nicaragua, and Costa Rica during 2021. These countries were selected because, in each of them, it was possible to locate a research dentist who agreed to participate in the study and take charge of collecting data and providing information on the situation of dentists in the pandemic context. Despite other countries being invited to participate in this study through several contacts, no answer was obtained, and we decided to carry this research with these participant countries.

The plan was to obtain a minimum quota sample of 200 dentists per country, to have 2200 participants in total. The research team decided to perform this type of sample considering the nature of the design, the scope of the findings, and the low possibility of carrying out a population study with statistically representative samples of dentists in the countries [23]. Thus, a nonprobabilistic selection was made until the required sample size was completed while still ensuring that the eligibility criteria were fulfilled: dentists actively practicing their profession, whose main activity is dental care or teaching practice, without a current diagnosis of psychological disorder (depression or anxiety), and who do not receive a retirement pension.

### 2.2. Data Collection Procedure

An online self-administered questionnaire managed through the Google Forms platform was used. To ensure an adequate sample, the survey link was distributed through digital media (Facebook groups, WhatsApp messages, emails, and institutional invitations) to several dental schools, dental associations, and personal messages to colleagues in participant countries (questionnaire available upon reasonable request). This form of survey distribution attempted to guarantee that only dentists answered this questionnaire. Data were collected from 1 June to 10 July 2021. Individuals who agreed to participate in the study by completing the survey and who met the eligibility criteria were included in the study.

The original Spanish questionnaire had three sections. The first was informed consent and three questions regarding the characteristics to identify the selection criteria. The second included the sociodemographic characteristics of the dentist: sex, age, country of origin, years of professional practice, highest academic degree achieved (bachelor, master, PhD), highest professional title achieved (general dentist, specialist), specialty domain, type of activity carried out (clinical care, teaching-research, both), weekly working hours, type of main remuneration (fixed salary, variable salary based on the activities carried out), and their perception of the pandemic’s economic impact on their income (none, mild, moderate, severe).

The third section measured labor well-being through the general labor well-being questionnaire (qBLG) proposed by Blanch et al. [9], which consists of 55 items divided into two factors/dimensions: psychosocial well-being and collateral effects. The first dimension consists of 42 items, grouped in turn into three specific factors: emotions (10 items), skills (10 items), and expectations (22 items). The collateral effects dimension consists of three specific factors: somatization (5 items), wear (4 items), and alienation (4 items). Response categories are given on a rating scale from 1 to 7 (a higher value indicates a higher intensity of the evaluated characteristic). Work well-being will be higher when the psychosocial well-being dimension has a higher score on the scale, and the collateral effects dimension has a lower score on the scale. Table S1 (Supplementary Material) explains in greater detail each of the proposed items and their score. This questionnaire was created to be administered to health professionals and was previously validated on a similar population, presenting psychometric properties that allow the study variable to be reproduced [9]. All of the factors showed high internal consistency, with Cronbach’s α values between 0.82 (alienation) and 0.96 (expectations). Results indicate that the qBLG questionnaire faithfully reproduces the structure of the proposed theoretical model [9]. The average time to complete the questionnaire was 5 min.

A pilot study was carried out on 110 dentists (10 per country). This process allowed for improving intelligibility, assessing time to completion, and evaluating internal consistency (understanding of the diverse questions for gathering information). The qBLG was tested, through Cronbach’s alpha (≥0.886), for each of the factors belonging to the two dimensions of the questionnaire. After this process, the application of the questionnaire was carried out in the study sample for the Latin American countries in a definitive way (more than 2000 individuals).

### 2.3. Statistical Analysis

Data processing and analysis were carried out using the statistical program SPSS v 21. Descriptive statistics were applied to the qualitative variables using frequency distribution tables. Correspondingly, measures of central tendency and dispersion were applied to the quantitative variables. The bivariate analysis of the work well-being score was performed according to the sociodemographic characteristics for which the Mann–Whitney U and Kruskal–Wallis tests were used, given that the displayed distribution was not normal. A multivariate linear regression analysis was performed: a hyper-hierarchical regression model to evaluate the joint effect of the explanatory variables on work well-being and the changes in the variance between each model (R2). Three models were created: Model 1 was adjusted according to sociodemographic variables such as sex, age, and country of origin. Model 2 incorporated the variables corresponding to the professional experience achieved (years of work practice and highest training achieved). Model 3 included the variables related to professional practice (dentistry activity, hours of professional practice, type of remuneration, and economic impact). The previous logarithmic transformation was performed on both dimensions of the work well-being variable since it did not display a normal distribution. A 5% significance level was established.

### 2.4. Ethics

The authors complied with the Declaration of Helsinki principles (2013). Informed consent and voluntary acceptance to participate in the study were requested. The study was approved by the Research Ethics Committee of the Faculty of Medicine of the National University of San Marcos (code 0112). Dentists agreed to an informed consent form linked to the beginning of the online form that had to be reviewed by the participant prior to completing the questionnaire. Participants’ anonymity was guaranteed at all times and the information obtained was kept completely confidential and used only for research purposes.

## 3. Results

In total, 2342 questionnaires were distributed and collected according to the answers provided by Google Forms. Out of those, 128 had to be discarded because there were some questions left blank or they registered an “unreal” value (despite our offering all of the information in the questionnaire). Therefore, the final sample consisted of 2214 dentists (response rate: 94.5%), mainly female (66.9%), with a higher participation of people aged between 35 and 49 years old. Eleven countries from Central and South America participated. The larger part of the sample was from Colombia (n = 323; 14.6%), followed by Ecuador (n = 314; 14.2%). The lowest participation of the sample was from Uruguay (3.1%) and Costa Rica (2.3%). General dentists constituted 38.3% (849) of the surveyed, while 7% had a PhD degree. The specialty with the highest percentage on the responses was “other specialty” (administration, ethics, and social sciences, among others), followed by orthodontics (n = 209; 15.9%). One hundred and ninety-two (14.6%) respondents reported having more than one specialty. On the other hand, a large portion of the participants practiced their profession for between 1 and 15 years (54.4%); 64.6% (1431 professionals) reported working in patient care, having 21 to 40 weekly work hours (44.3%; n = 980) and a variable salary/income per month (n = 1491; 67.3%). Of the participating dentists, 68.6% reported experiencing a moderate or severe impact on their income during the SARS-CoV-2 pandemic (COVID-19) (n = 997; 45%), those who considered the impact to be severe were less (23.6%), and only 11.3% (n = 250) stated that they had no impact whatsoever (Table 1).

When analyzing the two dimensions of work well-being, a psychosocial well-being score of 233.64 (±40.19) and collateral effects of 45.03 (±20.06) were found. Psychosocial well-being was higher and the collateral effect was lower in male dentists, in residents of Mexico and Central America, in those who had 31 years or more of professional practice, in those who performed both dental activities (teaching and research and clinical care) and when the economic impact of the pandemic was from none to slight (*p* < 0.05) (Table 2).

Somatization was frequently expressed through back pain (88.2%), muscle tension (87.2%) and, to a lesser extent, digestive disorders (64.3%), obtaining the highest average scores: 4.37 (±2.01) and 4.29 (±2.01). Wear due to physical exhaustion occurred in 87.5% of the dentists with a score of 4.11 (±1.99). The next highest value was work overload (82.3%), having an average score of 3.86 (±2.05). Bad mood and low professional fulfillment were frequent, representing 71.5% and 66.7% respectively (Table 3).

Table 4 shows the analyses for the two dimensions of work well-being, psychosocial well-being, and collateral effects, according to the origin country of participants. The mean score for psychological well-being was higher for dentists from Mexico, Costa Rica, and Ecuador, and the mean score for collateral effects was higher for individuals from Argentina, Chile, and Paraguay. Multiple comparison tests were carried out (post hoc) to observe statistically significant differences between several pairs of countries. Fifty-five possible comparison pairs were obtained for both dimensions of work well-being. For psychosocial well-being, 13 statistically significant differences between pairs of countries were found and in the case of collateral effects, 12 statistically significant differences were observed for pairs of countries (Supplementary material: Tables S2 and S3, Figures S1 and S2).

According to the multivariate analysis of psychosocial well-being of dentists, three models that were significant as a whole can be observed (Table 5). Model 3, which incorporates all the variables, has the highest determination coefficient (R2) = 0.116, generating a significant change (change in R2 = 0.072 *p* < 0.001). Age, evaluated simultaneously with the rest of the variables, does not show a significant association in any of the three models.

The multivariate analysis of the collateral effects dimension is significant in the three models. Model 3 has the highest determination coefficient (R2 = 0.084), generating a significant change (change R2 = 0.042 *p* < 0.001). When analyzing all the variables together, age and highest training achieved were not found to be significantly associated (Table 6).

## 4. Discussion

### 4.1. Possible Explanations for the Study Findings

In March 2020, the World Health Organization (WHO) released a statement characterizing the COVID−19 outbreak as a pandemic [24]. Drastic changes have been seen in the general well-being of the population, but the impact on the work well-being of dentists has not been extensively studied, much less in Latin American countries. Few studies have been published regarding the impact of COVID-19 on dentists in Central and South America [13,25], the largest number of publications being from countries in Europe and Asia [4,18,21,26,27,28,29,30,31,32,33]. Most of the published studies were carried out in 2020. Although few publications allow us to compare the evolution of the pandemic impact on the dentistry profession, we can attempt to show the outlook after a year and a half following the start of the pandemic.

This study has data from eleven Latin American countries, being the only one that incorporates so many countries in the region for 2021, gathering a total of 2214 surveys of oral health professionals. The study by León-Manco et al. is the most similar to this research study. It was carried out in the first six months of the pandemic, included various countries in Latin America and the Caribbean, had a total of 2036 surveys of dentists and dentistry students in the region, and evaluated the stress perceived due to the pandemic. These authors found a significant impact on the financial situation, career prospects, and personal lives of dentists and career students. Despite including a nongraduate population, this study proves that even in the initial stages of the pandemic there was already a considerable impact on important aspects of both personal and work life. Additionally, one year after this analysis it can be observed that this stress remains within the Latin American population [13]. Another complementary analysis carried out in Latin America and the Caribbean involving 1195 dentists between May and August 2021 found that the subjective perception of well-being had an average score of 56.83 out of a maximum possible score of 100 (maximum well-being) [14].

There are other studies such as that by Dávila-Torres et al., which was carried out in Ecuador between May and June 2020. This study evaluated the existence of anxiety caused by the pandemic and concluded that the Ecuadorian dentistry population experienced a state of moderate anxiety as a consequence of the pandemic. In accordance with our study, the results displayed greater female participation (74%) [25], which concurs with the results of various studies [26,29,31,33], and could be explained by the feminization that has been occurring in the profession for several decades.

The labor well-being of dentists is analyzed considering two dimensions: psychosocial well-being and collateral effects. A higher collateral effect suggests a lower well-being in dentists [9]. Somatization, wear, and alienation were the collateral effects evaluated. The ones that occurred most frequently were back pain and muscle tension as a consequence of the somatization experienced by dentists, as well as physical exhaustion and work overload within the wear component. However, these findings should not be attributed exclusively to the COVID-19 pandemic since dentists are prone to musculoskeletal disorders resulting from inadequate work ergonomics [34].

While this study was taking place, restrictions on the practice of dentistry had already decreased and most of the dentists were performing clinical activities. Even though a large percentage of the dentists were already vaccinated, the stress of working in the patient’s mouth with the high risk of contagion and with all of the biosafety equipment (aprons, double masks, and face shields, among others) makes clinical management more complex, which may explain the perception of higher overload and physical exhaustion, as well as the ailments reported. A Romanian study conducted on 83 dentists analyzed preventive dentistry behaviors related to the fear of COVID-19 contagion [35]. The main findings complement our results, considering the stress and anxiety that presented for dental health workers who had to adapt to all the dental practices in private and public health services. One study, conducted on 5370 dentists in Colombia, shows that COVID-19 has strongly impacted the practice of dentistry, leading to changes in clinical activities and career prospects [36]. However, although the contagion risk perception was high, self-reported contagion was very low and biosafety measures compliance was high. That situation means that the dentistry practice is safe and there is a need for policies and strategies for improving the quality of labor life of dentists, considering their actual situation. In addition, the findings raise an important question regarding the dentists working in hospitals. Conceivably, the workload of such dentists was extremely high, especially in the first months of the pandemic, because most private practices were closed. Complementary qualitative analyses should be conducted on those dentists involved in hospitals and health centers to understand the changes during the pandemic in labor well-being and its relationship with physical, mental, and psychosocial health.

In the present study, we found low professional fulfillment, which could have been caused by the situation of uncertainty created by the pandemic because, in spite of dentistry activities having restarted, patients still appear to be reluctant and consult almost only for emergency situations out of fear of contagion. Similarly, owing to biosafety protocols and capacity restrictions, dentists have reduced the number of patients they see per day. This altogether produces an economic impact and brings about the perception that the profession does not allow them to meet their economic needs. A study in Iranian dentists showed that 57% could face financial problems in the future [4]. Similarly, in studies carried out in Poland, Turkey, and Romania, a significant decrease in the income of dentists was found during the pandemic [35,37].

When stress exceeds the individual’s coping capacity, it can lead to wear. High levels of stress, exhaustion, anxiety, and psychological distress were associated with decreased personal well-being [38].

Labor well-being was lower in women, which concurs with various studies [13,28,31,33], where a higher level of stress and anxiety was reported in this group. Mekhermar et al. found higher levels of anxiety and stress in women and in middle-aged individuals [33]. Gasparro et al., in Italy, found that women were more afraid of COVID-19 and showed more depressive symptoms than men [30]. In Spain, women, in comparison to men, reported being more concerned about contracting COVID-19 for not having adequate protection measures, as well as being possible SARS-CoV-2 transmitters for patients [29]. Circumstances such as stress, depression, and concern about contagion lead to a decrease in well-being, which is evident in these groups. According to a recent study, perceived well-being was lower in Latin American female dentists compared to males [14]. In the Latin American background, women tend to have more responsibility in the household, which results in more work overload. Similarly, the fear of being infected and infecting their patients and their family contributes to a higher level of worry, stress, and even less well-being [29,30].

Regarding age, in the psychosocial well-being dimension, no significant differences were found. However, they were found in the collateral effect dimension, where a lower collateral effect and consequently a higher perceived well-being were evidenced in the older age groups and in individuals with more years of professional practice. This could be a result of this group being more mature and having more professional stability, as opposed to the younger ones, many of whom are just starting their professional careers and who have not reached a level of employment and economic stability that could provide them with a certain degree of peace of mind to face a limiting situation such as the COVID-19 pandemic. At the Latin American level, Garcés-Elías et al. [14] and Ortega-López et al. [15], respectively, found greater well-being and a lower level of perceived stress in older individuals, which is consistent with our results. When interpreting the results of people over 60 years of age showing less stress and anxiety, Mekhermar et al., in Germany, attributed it, among other aspects, to the fact that the elderly have gone through strong experiences in the past such as wars, crises, and pandemics, together with the fact that they spend less time on social networks exposed to ample information concerning the pandemic, which can ultimately be a stressor [33].

Labor well-being was lower in those who worked in clinical care compared to those who work in teaching and research. In a study carried out in the United Kingdom at the beginning of the pandemic, it was found that dentists who worked in clinical care in hospitals were more stressed compared to those who performed other types of activities [18]. It is very known that dentists in clinical practice face a higher risk of contracting SARS-CoV-2 and thus, they face all the consequences this entails, such as fear of becoming infected, taking the infection from the clinic to their families, being quarantined if they become infected, and even closing their clinics until the number of COVID-19 cases drops significantly [39]. Another consideration that favors the idea of higher well-being in dentists who work in teaching and research is the possibility of having an economic income, product of this activity, which grants more stability and economic ease. Furthermore, these activities provided the opportunity to keep working; despite the restrictions in most countries, they continued remotely, which allowed dentists to remain active and lessened their chances of experiencing feelings of anxiety, anguish, and worry. In the United Kingdom, it was found that dentists who were not working showed more depressive symptoms compared to those who did work [32]. The level of stress linked to the concern about their professional future in Turkish dentists was higher in those who worked in private practices compared to those who worked in hospitals [31]. To match this with our results, those who work in teaching and research in the Latin American context work mainly in university centers or hospitals, with a fixed economic income that may have been less affected in comparison with income from clinical care practice.

When performing the multivariate analysis, the three models presented were significant. It can be seen that in the adjusted model where all the variables were incorporated, age was not significant in any dimension. Some regional differences amongst dentists were found and labor well-being tended to be better in Central American countries and Mexico than in South American countries, which allows us to deduce the resemblance of the South American context.

### 4.2. Strength and Weakness of This Study

Regarding the instrument used, it is important to point out that, although it was validated in a sample of 1252 professionals from the health and education areas who worked in hospitals and universities [9], it has not been standardized internationally in other contexts and samples. That is the reason why the researchers carried out a pilot test in all the countries included in the present study, to support the decision to use by means of the labor components that include the questionnaire. However, not having data collected before the pandemic using this same questionnaire is a limitation, as there is no point of comparison to know if the work well-being of Latin American dentists has improved or worsened. In addition, most of the current results, obtained by using other questionnaires, concern the initial stages of the pandemic and are placed in geographical and sociocultural contexts that may be very different from the Latin American one.

Concerning the wear scale that belongs to the collateral effects dimensions, the information provided is not enough to consider if the participants are at risk of burnout syndrome. For that purpose, specific questionnaires are strongly recommended. For instance, the Copenhagen Burnout Inventory was validated in health care workers during the COVID-19 pandemic [40], and one study conducted in UK dentists considered the Oldenburg Burnout Inventory [38]. Further research could elucidate specific factors and conditions related to the burnout syndrome in Latin American Dentists.

The results of this study should be interpreted considering the nature and limitations of cross-sectional studies that use self-report questionnaires. Although it is not possible to determine causality, important associations and relationships between the variables can be established in this type of design considering bivariate and multivariate analyses. Precisely this type of study has an interest in exploring unknown or not studied in-depth situations. From a public health perspective, the study of self-perceptions in different social groups considering social phenomena constitutes broad measures transcending restrictive biomedical views in health research.

Owing to the lack of updated lists in the different countries of dentists registered in government databases, it was necessary to carry out a quota sampling and this means that the population size of each country was not considered. The results cannot be inferred from the total population of dentists, since a probabilistic sample was not possibly obtained. At this point, it is important to mention that, at the beginning of the study, we had planned to have a minimum sample of 200 participants for each country; however, in the final sample, some countries surpassed the participation quota and, on the contrary, others such as Uruguay and Costa Rica had the lower participation. The research team decided to keep this sample. Possible reasons for this low participation lie in the low motivation of the dentists’ associations and their reluctance to complete the online questionnaire, among other circumstances related to the fieldwork in each country. This situation did not affect the general findings shown in this study because the statistical power considering the nature of the main study variables was greater than 80%, as calculated by EPIDAT 3.1 (free distribution program developed by Dirección Xeral de Saúde Pública da Consellería de Sanidade, Xunta de Galicia, with the support of the Pan American Health Organization (PAHO–WHO) and the CES University of Colombia.

Considering these limitations, this study provides initial evidence of how the different measures that the governments implemented to control the pandemic affected these professionals who were studied, and it is the starting point for other studies that want to assess occupational well-being, a very important aspect to address since at least one- third or one-half of their time is spent at work.

### 4.3. Scope and Research Recommendations Derived of This Study

This research offers a general panorama of the general labor well-being of Latin American dentists in the context of the COVID-19 pandemic. It is important to emphasize that the findings of this research should be compared while always keeping in mind that each country has a different political and socioeconomic context and that the perceived impact of the pandemic in each varies according to the policies that have been deployed, the moment of implementation, the biosafety protocols, social groups, and the population responsibility to comply [41]. It should kept in mind that although most countries adopted the recommendations provided by the Pan American Health Organization (PAHO) [42], some assumed specific measures of a governmental nature. For example, the Ministry of Health and Social Protection of Colombia established the document entitled: Biosafety guidelines for the provision of services related to oral health care during the period of the SARS-CoV-2 (COVID-19) pandemic [43]. The Ministry of Health of Argentina published some recommendations for the dental practice [44], which were adjusted following WHO and PAHO recommendations. Other documentary studies should explore the adaptation of biosafety protocols proposed by international organizations, and the implementation of other restrictive measures and their acceptance by dental health care professionals.

The results obtained open the need to carry out individual research for each of the Latin American countries and to establish comparative studies through strong samples for each of them. For that purpose, exploring specific topics related to the impact of the pandemic in personal, familiar, and social lives by incorporating new contextual variables is recommended.

Similarly, dental specialties were not equally touched by the pandemic. In our study, the participation of postgraduate dentists (clinical, MSc, and PhD) in the sample was 61.7%. Further research through quantitative, qualitative, and mixed methods should be conducted in specific clinical and other specialties, and by following other labor, physical, mental, and psychosocial indicators that permit an explanation of the impact of the COVID-19 pandemic in dental health care workers.

## 5. Conclusions

This study showed the impact of the COVID-19 pandemic, a year and a half after it began, on the labor well-being of Latin American dentists. The multivariate model displayed the interaction of the latter with social characteristics such as sex, country of origin, years of professional practice, type of dentistry activity, type of remuneration, and economic impact of the pandemic. According to the findings, epidemiological surveillance systems should be implemented to obtain reliable data on the employment situation and physical, mental, and psychosocial health indicators of dental health care workers. Similarly, strategies to promote mental health and prevent stress should be carried out considering the social reality of dentists in each Latin American country. Lastly, considering the dynamic and evolution of the pandemic, the evaluation of public policies on occupational health and safety should be achieved in the short, medium, and long term.

## Figures and Tables

**Table 1:** Characteristics of the participating dentists (n = 2214).

Variables	N	%
***Sex***		
Males	733	33.1
Females	1481	66.9
***Age (years)***		
20–34	796	36.0
35–49	856	38.7
≥51	562	25.4
***Country of Origin***		
Peru	293	13.2
Chile	163	7.4
Mexico	186	8.4
Colombia	323	14.6
Argentina	206	9.3
Ecuador	314	14.2
Uruguay	69	3.1
Venezuela	237	10.7
Paraguay	205	9.3
Nicaragua	168	7.6
Costa Rica	50	2.3
***Clinical experience (years)***		
≤15	1204	54.4
16–30	745	33.6
≥31	265	12.0
***Academic education***		
General dentist	849	38.3
Clinical specialist	794	35.9
MSc	417	18.8
PhD	154	7.0
***Specialty***		
Endodontics and cariology	124	9.5
Maxillofacial Surgery	154	11.7
Prosthodontics	46	3.5
Periodontics and implantology	102	7.8
Pediatric dentistry	177	13.5
Orthodontics	209	15.9
Forensic dentistry	5	0.4
Radiology	9	0.7
Public health	77	5.9
Other	217	16.5
More than one	192	14.6
***Dental activity***		
Teaching/Research	139	6.3
Clinical assistance	1431	64.6
Both	644	29.1
***Weekly working hours***		
1–20	578	26.1
21–40	980	44.3
≥41	656	29.6
**Type of salary (monthly)**		
Fixed	723	32.7
Variable	1491	67.3
**Economic impact of the pandemic**		
None	250	11.3
Mild	444	20.1
Moderate	997	45.0
Severe	523	23.6

**Table 2:** Psychosocial well-being and total score for collateral effects according to characteristics of the participants.

Variables	Psychosocial Well-Being (42–294)				Total Score for Collateral Effects (13–91)			
	Mean	SD	Median	*p*-Value	Mean	SD	Median	*p*-Value
***Sex***								
Males	241.23	36.3	246	<0.001	40.43	18.62	37	<0.001
Females	229.87	41.5	236		47.3	20.36	47	
***Age (years)***								
20–34	233.18	40.8	239	0.578	46.50 ^a^	19.97	46	<0.001
35–49	233.1	39.9	240		46.08 ^a^	20.29	44	
≥51	235.1	39.9	240		41.32 ^b^	19.41	38	
**Country of origin**								
South America	231.23	40.6	236.5	<0.001	45.48	20.08	44	0.022
Mexico and Central America	244.41	36.7	249		42.97	19.86	40	
***Clinical experience (years)***								
≤15	232.22 ^a^	41.2	238	0.039	46.92 ^a^	20.06	46	<0.001
16–30	234.14 ^ab^	38.2	240		44.34 ^b^	19.82	42	
≥31	238.66 ^b^	40.9	243		38.36 ^c^	19.23	34	
***Academic education***								
General dentist	227.30 ^a^	40.9	232	<0.001	46.04	19.93	45	0.143
Clinical specialist	236.09 ^b^	39.9	242		44.92	20.28	43	
MSc	241.18 ^b^	36.7	245		43.95	19.73	40	
PhD	235.47 ^b^	42.6	248		42.89	20.37	40	
**Specialty**								
Endodontics and cariology, Maxillofacial Surgery, Prosthodontics, Periodontics and implantology	237.79	39	244.5	0.581	44.07	20.43	40	0.466
Pediatric dentistry, Orthodontics, Forensic dentistry, Radiology, Public health, Other	237.84	38.8	244		44.11	19.89	41	
More than one	234.22	41.7	240.5		46.05	20.46	47	
***Dental activity***								
Teaching/Research	233.85 ^ab^	42.7	242	<0.001	47.57 ^ab^	19.46	47	0.015
Clinical assistance	230.33 ^a^	40.7	235		45.54 ^a^	19.99	44	
Both	240.93 ^b^	37.5	247		43.34 ^b^	20.25	40	
***Weekly working hours***								
1 to 20	230.75	41.3	236	0.143	41.85 ^a^	19.6	39	<0.001
21 to 40	234.84	40.3	241		44.38 ^b^	19.84	42	
≥31	234.38	38.9	241		48.80 ^c^	20.22	48	
**Type of salary**								
Fixed	231.58	40.5	238	0.095	46.09	20.32	45	0.092
Variable	234.63	40.1	240		44.51	19.92	43	
**Economic impact of the pandemic**								
None	245.16 ^a^	39.5	254.5	<0.001	42.34 ^a^	20.77	40	<0.001
Mild	243.22 ^a^	36.5	248		42.67 ^a^	18.97	40	
Moderate	235.98 ^b^	36.8	240		44.37 ^a^	19.82	42	
Severe	215.53 ^b^	43.8	219		49.57 ^b^	20.39	51	

Different letters indicate statistically significant differences. The values in parentheses for the psychosocial well-being and the collateral effects indicate the minimum and maximum score to be reached by the dimension.

**Table 3:** Specific collateral effects in the participants.

Collateral Effects	Mean	SD	Median	No Presence (1)	Presence (2–7)
***Somatization***					
Digestive disorders	2.96	1.973	2	790 (35.7%)	1424 (64.3%)
Headache	3.26	1.972	3	60 (27.3%)	1610 (72.7%)
Insomnia	3.01	1.986	2	753 (34.0%)	1461 (66.0%)
Backache	4.37	2.011	5	261 (11.8%)	1953 (88.2%)
Muscle tensions	4.29	2.014	5	284 (12.8%)	1930 (87.2%)
***Wear***					
Work overload	3.86	2.051	4	392 (17.7%)	1822 (82.3%)
Emotional stress	3.78	2.085	4	428 (19.3%)	1786 (80.7%)
Physical exhaustion	4.11	1.995	4	276 (12.5%)	1938 (87.5%)
Mental saturation	3.81	2.071	4	409 (18.5%)	1805 (81.5%)
***Alienation***					
Bad mood	3.04	1.88	3	632 (28.5%)	1582 (71.5%)
Low professional achievement	2.97	1.902	2	738 (33.3%)	1476 (66.7%)
Depersonalized treatment	2.67	1.857	2	913 (41.2%)	1301 (58.8%)
Frustration	2.91	1.91	2	755 (34.1%)	1459 (65.9%)

The presence of different collateral effects was defined with the responses from 2 to 7 according to the questionnaire.

**Table 4:** Total scores for collateral effects according to the origin country of participants.

Origin Country	Psychosocial Well-Being (42–294) *			Collateral Effects (13–91) *		
	Mean	SD	Median	Mean	SD	Median
Peru	235.97	37.64	240	41.45	18.55	39
Chile	233.92	36.86	239	49.67	20.87	52
Mexico	245.02	36.33	249	44.87	20.67	40
Colombia	231.11	41.36	237	43.72	19.57	43
Argentina	224.08	43.39	232	52.4	19.43	55
Ecuador	241.03	39.26	247	46.55	20.44	44.5
Uruguay	230.26	35.01	235	47.33	18.8	48
Venezuela	225.66	42.38	230	39.41	18.72	36
Paraguay	221.46	40.78	222	48.52	21.08	47
Nicaragua	244.73	35.5	248	40.01	18.44	37
Costa Rica	241.06	42.3	252	45.86	20.51	40

* Kruskal–Wallis test: *p*-value < 0.001 for both dimensions (statistically significant differences when all countries in the sample are considered).

**Table 5:** Multiple linear regression model of the psychosocial well-being of dentists (n = 2214).

Variables	Determination Coefficient (R2)	Change in R2	*p*-Value of Change R2	Constant	Nonstandardized Regression Coefficient	Standardized Regression Coefficient	95% Confidence Interval		*p*-Value	*p*-Value Model
***Model 1***										<0.001
Sex (F)	0.03	0.03	<0.001	0.527	−0.034	−0.105	−0.047	−0.02	<0.001	
Age					0.008	0.042	0	0.016	0.056	
Country of origin (México—Central America)					0.052	0.132	0.035	0.069	<0.001	
***Model 2***										
Sex (F)	0.044	0.014	<0.001	0.515	−0.03	−0.094	−0.044	−0.017	<0.001	<0.001
Age					−0.013	−0.064	−0.026	0.001	0.072	
Country of origin (México—Central America)					0.052	0.132	0.035	0.068	<0.001	
Clinical experience (years)					0.018	0.083	0.003	0.033	0.016	
Academic education					0.019	0.113	0.011	0.026	<0.001	
***Model 3***										
Sex (F)	0.116	0.072	<0.001	0.551	−0.027	−0.085	−0.04	−0.015	<0.001	<0.001
Age					−0.005	−0.025	−0.018	0.008	0.465	
Country of origin (México—Central America)					0.048	0.122	0.032	0.064	<0.001	
Clinical experience (years)					0.016	0.072	0.001	0.03	0.031	
Academic education					0.014	0.086	0.007	0.021	<0.001	
Dental activity					0.016	0.058	0.005	0.027	0.006	
Weekly working hours					−0.007	−0.034	−0.015	0.001	0.104	
Type of salary (variable)					0.048	0.147	0.034	0.062	<0.001	
Economic impact of the pandemic					−0.046	−0.28	−0.053	−0.039	<0.001	

**Table 6:** Multiple linear regression model of collateral effects in dentists. (n = 2214).

Variables	Determination Coefficient (R2)	Change in R2	*p*-Value of Change R2	Constant	Nonstandardized Regression Coefficient	Standardized Regression Coefficient	95% Confidence Interval		*p*-Value	*p*-Value Model
***Model 1***										<0.001
Sex (F)	0.036	0.036	<0.001	0.513	0.048	0.147	0.035	0.061	<0.001	
Age					−0.021	−0.104	−0.029	−0.012	<0.001	
Country of origin (México—Central America)					−0.025	−0.062	−0.042	−0.008	0.004	
***Model 2***										
Sex (F)	0.042	0.006	<0.001	0.512	0.046	0.141	0.033	0.06	<0.001	<0.001
Age					0	−0.001	−0.014	0.014	0.975	
Country of origin (México—Central America)					−0.025	−0.063	−0.042	−0.008	0.003	
Clinical experience (years)					−0.029	−0.131	−0.044	−0.014	<0.001	
Academic education					0	0.001	−0.007	0.007	0.977	
***Model 3***										
Sex (F)	0.084	0.042	<0.001	0.464	0.049	0.15	0.036	0.062	<0.001	<0.001
Age					−0.001	−0.003	−0.014	0.013	0.927	
Country of origin (México—Central America)					−0.02	−0.05	−0.037	−0.003	0.018	
Clinical experience (years)					−0.026	−0.119	−0.041	−0.012	<0.001	
Academic education					0	0.002	−0.007	0.008	0.934	
Dental activity					−0.014	−0.051	−0.026	−0.003	0.017	
Weekly working hours					0.031	0.153	0.023	0.04	<0.001	
Type of salary (variable)					−0.028	−0.085	−0.042	−0.013	<0.001	
Economic impact of the pandemic					0.027	0.16	0.019	0.034	<0.001	

## Data Availability

Not applicable.

## Supplementary Materials

The following supporting information can be downloaded at: https://www.mdpi.com/article/10.3390/ijerph19106317/s1, Table S1: Scales and items of the Questionnaire of General Labor Well-Being (qBLG); Table S2: Multiple pairwise comparisons for the origin country of participants and the scores of psychosocial well-being; Figure S1: Multiple pairwise comparisons for the origin country of participants and the scores of psychosocial well-being; Table S3: Multiple pairwise comparisons for the origin country of participants and the scores of collateral effects; Figure S2: Multiple pairwise comparisons for the origin country of participants and the scores of collateral effects.
