# Supplementary material for: General Labor Well-Being in Latin American Dentists during the COVID-19 Pandemic
Source: Int J Environ Res Public Health. 2022 May 23;19(10):6317. doi: 10.3390/ijerph19106317 (PMC9141737; doi:10.3390/ijerph19106317)
Supplement: Supplementary file 1 [file ijerph-19-06317-s001.zip › ijerph-1679238-supplementary.pdf]

Table S1. Scales and items of the Questionnaire of General Labor Well-Being (qBLG)*

**Factor: Psychosocial Well-Being**									
	**Emotions Scale**								
**Currently at my job I feel:**									
	Dissatisfaction	1	2	3	4	5	6	7	Satisfaction
	Insecurity	1	2	3	4	5	6	7	Security
	Restlessness	1	2	3	4	5	6	7	Tranquility
	Impotence	1	2	3	4	5	6	7	Potency
	Discomfort	1	2	3	4	5	6	7	Well-being
	Distrust	1	2	3	4	5	6	7	Trust
	Uncertainty	1	2	3	4	5	6	7	Certainty
	Confusion	1	2	3	4	5	6	7	Clarity
	Hopelessness	1	2	3	4	5	6	7	Hope
	Difficulty	1	2	3	4	5	6	7	Facility
	**Skills Scale**								
**Currently at my job I feel:**									
	Insensitivity	1	2	3	4	5	6	7	Sensitivity
	Irrationality	1	2	3	4	5	6	7	Rationality
	Incompetence	1	2	3	4	5	6	7	Competence
	Immorality	1	2	3	4	5	6	7	Morality
	Badness	1	2	3	4	5	6	7	Goodness
	Failure	1	2	3	4	5	6	7	Success
	Incapacity	1	2	3	4	5	6	7	Capacity
	Pessimism	1	2	3	4	5	6	7	Optimism
	Inefficacy	1	2	3	4	5	6	7	Efficacy
	Uselessness	1	2	3	4	5	6	7	Usefulness
**Expectations Scale**									
**In my career:**									
My motivation for working	Going down	1	2	3	4	5	6	7	Going up
My identification with the values of the organization	Going down	1	2	3	4	5	6	7	Going up
My professional performance	Going down	1	2	3	4	5	6	7	Going up
My ability to manage my workload	Going down	1	2	3	4	5	6	7	Going up
The quality of my working conditions	Going down	1	2	3	4	5	6	7	Going up
My professional self-esteem	Going down	1	2	3	4	5	6	7	Going up
The cordiality in my work environment	Going down	1	2	3	4	5	6	7	Going up
Reconciling my work with my private life	Going down	1	2	3	4	5	6	7	Going up
My confidence in my professional future	Going down	1	2	3	4	5	6	7	Going up
My quality of life at work	Going down	1	2	3	4	5	6	7	Going up
The meaning of my work	Going down	1	2	3	4	5	6	7	Going up
My compliance with management guidelines	Going down	1	2	3	4	5	6	7	Going up
My mood at work	Going down	1	2	3	4	5	6	7	Going up
My career advancement opportunities	Going down	1	2	3	4	5	6	7	Going up
My sense of security at work	Going down	1	2	3	4	5	6	7	Going up
My participation in the decisions of the organization	Going down	1	2	3	4	5	6	7	Going up
My job satisfaction	Going down	1	2	3	4	5	6	7	Going up
My professional fulfillment	Going down	1	2	3	4	5	6	7	Going up
The level of excellence in my organization	Going down	1	2	3	4	5	6	7	Going up
My professional effectiveness	Going down	1	2	3	4	5	6	7	Going up

My commitment at work	Going down	1	2	3	4	5	6	7	Going up
My professional skills	Going down	1	2	3	4	5	6	7	Going up
**Factor: Collateral Effects**									
**Somatization Scale**									
**Currently, because of my work, I feel:**									
Digestive disorders	Never	1	2	3	4	5	6	7	Always
Headache	Never	1	2	3	4	5	6	7	Always
Insomnia	Never	1	2	3	4	5	6	7	Always
Backache	Never	1	2	3	4	5	6	7	Always
Muscle tensions	Never	1	2	3	4	5	6	7	Always
**Wear Scale**									
**Currently, because of my work, I feel:**									
Work overload	Never	1	2	3	4	5	6	7	Always
Emotional stress	Never	1	2	3	4	5	6	7	Always
Physical exhaustion	Never	1	2	3	4	5	6	7	Always
Mental saturation	Never	1	2	3	4	5	6	7	Always
**Alienation Scale**									
**Currently, because of my work, I feel:**									
Bad mood	Never	1	2	3	4	5	6	7	Always
Low professional achievement	Never	1	2	3	4	5	6	7	Always
Depersonalized treatment	Never	1	2	3	4	5	6	7	Always
Frustration	Never	1	2	3	4	5	6	7	Always

* The original qBLG was validated in Spanish. The factors and scales were translated from Spanish to English for didactic purposes. Content/Construct validation is recommended for research outside Spanish of Latin American contexts.

Table S2. Multiple pairwise comparisons for the origin country of participants and the scores of psychosocial well-being

Sample 1-Sample 2	Pairwise comparisons by origin country: Psychosocial well-being*				
	Test statistic	Std. Error	Std. Test statistic	Sig.	Adj. Sig.
Paraguay-Argentina	64.838	63.063	1.028	.304	1.000
Paraguay-Venezuela	80.514	60.972	1.321	.187	1.000
Paraguay-Uruguay	104.088	88.969	1.170	.242	1.000
Paraguay-Colombia	164.192	57.083	2.876	.004	.221
Paraguay-Chile	191.542	67.084	2.855	.004	.237
Paraguay-Peru	222.704	58.206	3.826	.000	.007
Paraguay-Ecuador	325.851	57.400	5.677	.000	.000
Paraguay-Costa Rica	-352.298	100.827	-3.494	.000	.026
Paraguay-Nicaragua	-371.900	66.526	-5.590	.000	.000
Paraguay-México	390.120	64.732	6.027	.000	.000
Argentina-Venezuela	-15.676	60.892	-.257	.797	1.000
Argentina-Uruguay	-39.250	88.915	-.441	.659	1.000
Argentina-Colombia	99.354	56.998	1.743	.081	1.000
Argentina-Chile	126.704	67.012	1.891	.059	1.000
Argentina-Peru	157.866	58.123	2.716	.007	.363
Argentina-Ecuador	-261.013	57.315	-4.554	.000	.000
Argentina-Costa Rica	-287.460	100.779	-2.852	.004	.239
Argentina-Nicaragua	-307.063	66.453	-4.621	.000	.000
Argentina-Mexico	325.282	64.658	5.031	.000	.000
Venezuela-Uruguay	23.574	87.444	.270	.787	1.000
Venezuela-Colombia	83.678	54.675	1.530	.126	1.000
Venezuela-Chile	111.028	65.047	1.707	.088	1.000
Venezuela-Peru	142.190	55.847	2.546	.011	.599
Venezuela-Ecuador	245.337	55.005	4.460	.000	.000
Venezuela-Costa Rica	-271.784	99.483	-2.732	.006	.346
Venezuela-Nicaragua	-291.386	64.471	-4.520	.000	.000
Venezuela-Mexico	309.606	62.619	4.944	.000	.000
Uruguay-Colombia	60.104	84.778	.709	.478	1.000
Uruguay-Chile	87.454	91.811	.953	.341	1.000
Uruguay-Peru	118.616	85.539	1.387	.166	1.000
Uruguay-Ecuador	221.763	84.992	2.609	.009	.499
Uruguay-Costa Rica	-248.210	118.722	-2.091	.037	1.000
Uruguay-Nicaragua	-267.813	91.403	-2.930	.003	.186
Uruguay-Mexico	286.032	90.106	3.174	.002	.083
Colombia-Chile	27.350	61.417	.445	.656	1.000
Colombia-Peru	58.512	51.573	1.135	.257	1.000
Colombia-Ecuador	-161.659	50.661	-3.191	.001	.078
Colombia-Costa Rica	-188.106	97.148	-1.936	.053	1.000
Colombia-Nicaragua	-207.709	60.807	-3.416	.001	.035

Sample 1-Sample 2	Pairwise comparisons by origin country: Psychosocial well-being*				
	Test statistic	Std. Error	Std. Test statistic	Sig.	Adj. Sig.
Colombia-Mexico	225.929	58.839	3.840	.000	.007
Chile-Peru	31.162	62.463	.499	.618	1.000
Chile-Ecuador	-134.309	61.712	-2.176	.030	1.000
Chile-Costa Rica	-160.756	103.342	-1.556	.120	1.000
Chile-Nicaragua	-180.359	70.280	-2.566	.010	.565
Chile-Mexico	-198.578	68.585	-2.895	.004	.208
Peru-Ecuador	-103.147	51.923	-1.987	.047	1.000
Peru-Costa Rica	-129.594	97.813	-1.325	.185	1.000
Peru-Nicaragua	-149.196	61.863	-2.412	.016	.873
Peru-Mexico	-167.416	59.930	-2.794	.005	.287
Ecuador-Costa Rica	-26.447	97.335	-.272	.786	1.000
Ecuador-Nicaragua	-46.050	61.104	-.754	.451	1.000
Ecuador-Mexico	64.270	59.147	1.087	.277	1.000
Costa Rica-Nicaragua	19.603	102.981	.190	.849	1.000
Costa Rica-Mexico	37.822	101.831	.371	.710	1.000
Nicaragua-Mexico	18.220	68.039	.268	.789	1.000

* Significance values were adjusted using the Bonferroni correction for various tests.

Figure S1. Multiple pairwise comparisons for the origin country of participants and the scores of psychosocial well-being*

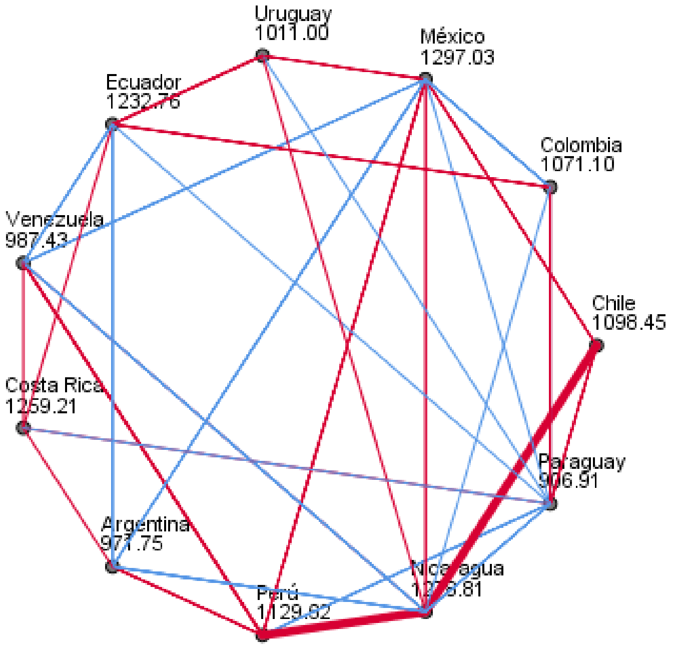

* Each node represents the rank mean of the sample from the country of origin
The blue lines represent the statistically significant differences between pairs of countries.

Table S3. Multiple pairwise comparisons for the origin country of participants and the scores of collateral effects.

Sample 1-Sample 2	Pairwise comparisons by origin country: Collateral effects*				
	**Test statistic**	**Std. Error**	**Std. Test statistic**	**Sig.**	**Adj. Sig.**
Venezuela-Nicaragua	-23.022	64.466	-.357	.721	1.000
Venezuela-Peru	67.891	55.842	1.216	.224	1.000
Venezuela-Colombia	139.197	54.671	2.546	.011	.599
Venezuela-Mexico	171.850	62.614	2.745	.006	.333
Venezuela-Costa Rica	-199.199	99.476	-2.002	.045	1.000
Venezuela-Ecuador	226.060	55.001	4.110	.000	.002
Venezuela-Uruguay	263.990	87.437	3.019	.003	.139
Venezuela-Paraguay	-285.210	60.967	-4.678	.000	.000
Venezuela-Chile	324.289	65.042	4.986	.000	.000
Venezuela-Argentina	417.999	60.888	6.865	.000	.000
Nicaragua-Peru	44.869	61.858	.725	.468	1.000
Nicaragua-Colombia	116.175	60.802	1.911	.056	1.000
Nicaragua-Mexico	148.828	68.034	2.188	.029	1.000
Nicaragua-Costa Rica	-176.177	102.973	-1.711	.087	1.000
Nicaragua-Ecuador	203.038	61.100	3.323	.001	.049
Nicaragua-Uruguay	240.967	91.397	2.637	.008	.461
Nicaragua-Paraguay	262.187	66.521	3.941	.000	.004
Nicaragua-Chile	301.266	70.275	4.287	.000	.001
Nicaragua-Argentina	394.977	66.448	5.944	.000	.000
Peru-Colombia	-71.306	51.569	-1.383	.167	1.000
Peru-Mexico	-103.959	59.926	-1.735	.083	1.000
Peru-Costa Rica	-131.308	97.805	-1.343	.179	1.000
Peru-Ecuador	-158.169	51.919	-3.046	.002	.127
Peru-Uruguay	-196.099	85.532	-2.293	.022	1.000
Peru-Paraguay	-217.319	58.202	-3.734	.000	.010
Peru-Chile	-256.397	62.458	-4.105	.000	.002
Peru-Argentina	-350.108	58.119	-6.024	.000	.000
Colombia-Mexico	32.653	58.835	.555	.579	1.000
Colombia-Costa Rica	-60.001	97.141	-.618	.537	1.000
Colombia-Ecuador	-86.863	50.657	-1.715	.086	1.000
Colombia-Uruguay	-124.792	84.772	-1.472	.141	1.000
Colombia-Paraguay	-146.012	57.079	-2.558	.011	.579
Colombia-Chile	185.091	61.413	3.014	.003	.142
Colombia-Argentina	-278.802	56.994	-4.892	.000	.000
Mexico-Costa Rica	-27.349	101.824	-.269	.788	1.000
Mexico-Ecuador	-54.210	59.142	-.917	.359	1.000
Mexico-Uruguay	-92.139	90.100	-1.023	.306	1.000
Mexico-Paraguay	-113.359	64.728	-1.751	.080	1.000
Mexico-Chile	152.438	68.580	2.223	.026	1.000
Mexico-Argentina	-246.149	64.653	-3.807	.000	.008
Costa Rica-Ecuador	26.861	97.327	.276	.783	1.000
Costa Rica-Uruguay	64.791	118.713	.546	.585	1.000
Costa Rica-Paraguay	86.011	100.819	.853	.394	1.000
Costa Rica-Chile	125.090	103.335	1.211	.226	1.000

Sample 1-Sample 2	Pairwise comparisons by origin country: Collateral effects*				
	Test statistic	Std. Error	Std. Test statistic	Sig.	Adj. Sig.
Costa Rica-Argentina	218.801	100.771	2.171	.030	1.000
Ecuador-Uruguay	-37.930	84.985	-.446	.655	1.000
Ecuador-Paraguay	-59.150	57.395	-1.031	.303	1.000
Ecuador-Chile	98.229	61.707	1.592	.111	1.000
Ecuador-Argentina	191.940	57.311	3.349	.001	.045
Uruguay-Paraguay	-21.220	88.963	-.239	.811	1.000
Uruguay-Chile	60.299	91.804	.657	.511	1.000
Uruguay-Argentina	154.010	88.908	1.732	.083	1.000
Paraguay-Chile	39.079	67.079	.583	.560	1.000
Paraguay-Argentina	132.790	63.059	2.106	.035	1.000
Chile-Argentina	-93.711	67.007	-1.399	.162	1.000

* Significance values were adjusted using the Bonferroni correction for various tests.

Figure S2. Multiple pairwise comparisons for the origin country of participants and the scores of collateral effects.*

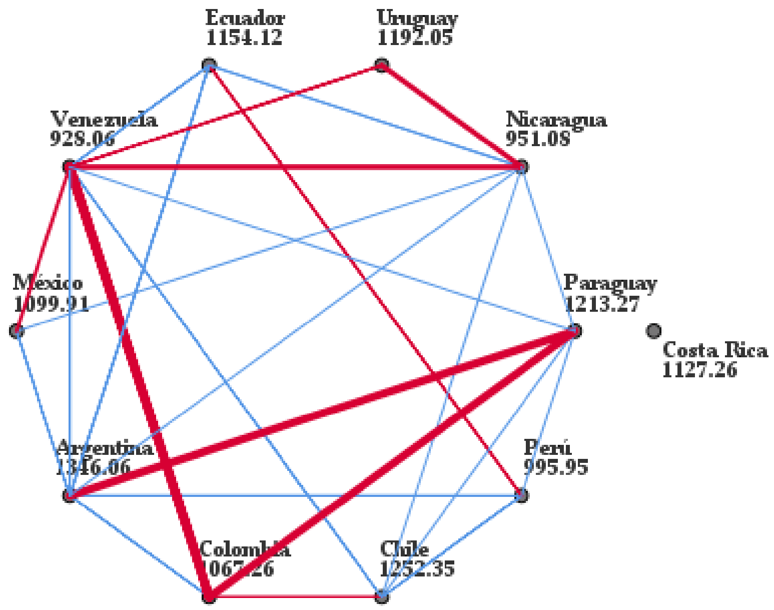

* Each node represents the rank mean of the sample from the country of origin
The blue lines represent the statistically significant differences between pairs of countries.
